# A Perspective on a Possible Relation Between the Psychopathology of the Schizophrenia/Schizoaffective Spectrum and Unconjugated Bilirubin: A Longitudinal Protocol Study

**DOI:** 10.3389/fpsyt.2018.00146

**Published:** 2018-04-23

**Authors:** João Gama Marques, Filipe Arantes-Gonçalves

**Affiliations:** ^1^Clínica Universitária de Psiquiatria e Psicologia Médica da Faculdade de Medicina da Universidade de Lisboa, Lisbon, Portugal; ^2^Hospital Júlio de Matos, Centro Hospitalar Psiquiátrico de Lisboa, Lisbon, Portugal; ^3^Clínica de Saúde Mental do Porto, Porto, Portugal; ^4^CliniPinel - Clínica de Psiquiatria, Psicoterapia e Psicanálise, Lisbon, Portugal

**Keywords:** schizophrenia, schizoaffective, psychoses, unconjugated bilirubin, unconjugated hyperbilirubinemia

## Abstract

Some authors suggest a relation between Unconjugated Bilirubin (UCB) plasma high levels and schizophrenia, as schizophrenia patients have been showing higher UCB levels when compared with other psychiatric patients and general population. These higher UCB levels have been already correlated with acute psychotic states, positive symptoms, and poor outcome in patients with schizophrenia. Schizophrenia and schizoaffective disorders share common symptoms but there aren't yet accepted biomarkers for their distinction. In our study protocol we propose an observational longitudinal study on a sample composed of two subgroups: patients with schizophrenia and patients with schizoaffective disorder. We will compare the UCB levels between groups, and search for a possible correlation with patient's psychopathology. For that purpose we will use nosological, psychopathological, neuropsychological, and psychosocial instruments. Thus we will be testing two different hypotheses: (1) Is UCB serum level a diagnosis indicator, with categorical distinction potential, between groups of patients with different psychotic disorders? (2) Is UCB serum level a severity indicator, with dimensional distinction potential, among groups of patients with the same psychotic disorder? We believe that UCB mean levels may contribute to some clarification of this controversy, as a potential biological indicator, facilitating the distinction between these two diagnostic categories and\or discriminating the dimensional severity among each of these psychotic conditions. Thus we may be opening a new opportunities for innovative and exciting biological psychiatry research regarding organic aspects in the schizophrenia spectrum.

## Introduction

Schizophrenia and several other psychiatric disorders share the complexity of their etiology, which reflects the interplay of different risk factors at multiple levels of analysis. The impact of psychosocial risk factors is well-established and neurobiological research is trying to define the underlying changes at molecular, cellular, anatomic or physiological levels that can support both the definition and the phenotypic presentation of those disorders, in a more integrative models.

In a recent review about the environment and susceptibility to schizophrenia, the authors stated that, in schizophrenia, the diversity of risk factors probably interacts in complex ways with the macrostructural environment, including the psychological, cultural, and socioeconomic context and he also points to the significant implications of the study of environmental factors to improve the explanatory power of neurodevelopmental models, the identification of causes and prevention of this complex disorder [1].

Schizophrenia is characterized by persistent cognitive, positive and negative symptoms typically beginning in youth, and brain alterations including dopaminergic dysregulation. Several pathophysiological models have been proposed, accumulating knowledge involving a neurodevelopmental imbalance in excitatory/inhibitory neurotransmitters, which could result from a variety of genetic, epigenetic and environmental causes, as well as pathophysiological processes such oxidative stress [2]. A dysregulation of the redox and glutamatergic systems due to genetic and early-life environmental risk factors could contribute to the anomalies of white matter in schizophrenia, ultimately impacting the patient's behavior via abnormal function of neural circuits [3].

## Unconjugated bilirubin: an underestimated neurotoxin?

High levels of UCB (the main product of heme catabolism), sometimes are observed in the newborn, resulting from a decreased erythrocyte survival and a deficient hepatic clearance [4]. A significantly increase in the prevalence of mental disorder among children with neonatal hyperbilirubinemia was found when compared to a control group [5]. Idiopathic unconjugated hyperbilirubinemia (Gilbert's syndrome) is a common genetic deficiency of the enzyme UDP-glucuronosyltransferase-1 that is found in a maximum of 10% of the general population [6], but may have the double of that prevalence among patients with schizophrenia [7].

The Gunn rat (a Wistar strain mutant), has a genetic deficiency in glucuronyl transferase [8] and has already been used as a schizophrenia animal model [9]. Some works showed that microglial activation due to UCB chronic toxicity could be an important causal factor in the behavioral neuropathological abnormalities of these rats [10] (after crossing the blood brain barrier). Other studies showed that the antipsychotic medication (e.g., aripiprazole, risperidone, or haloperidol) effect on the Gunn rats' behavior is quite similar to those verified in humans with schizophrenia, enhancing social behaviors (e.g., isolation) [11]. The physiopathology underneath is related with UCB's insult to glial cells, leading to glutamate secretion and release of pro-inflammatory cytokines that may influence gliogenesis and neurogenesis, with secondary deficit in learning and memory; while glutamate metabolism dysregulation is consistent with schizophrenia neuropathology [12].

## Unconjugated bilirubin: a biomarker candidate for acute psychosis?

Patients with schizophrenia have already shown a significantly higher frequency of UCB mean levels when compared with patients with bipolar disorder [13, 14]. This kind of evidence suggests that UCB high levels may have a potential role as an indicator in the categorical distinction of among different psychiatric diagnosis.

In the other hand, patients suffering from schizophrenia frequently presented with higher than expected plasma UCB concentration, especially when acutely ill and admitted to the hospital [15]. Some authors suggested that the association of hyperbilirubinemia and schizophrenia disorders is stronger in acute psychosis episodes [16, 17]. Indeed, patients with schizophrenia with higher rates of hyperbilirubinemia presented a positive correlation between bilirubin levels and psychosis severity, namely higher scores on the positive and negative symptoms (PANSS) [18–20]. Besides this correlation with acute psychosis, it might also represent a poor outcome for the schizophrenia patients with idiopathic unconjugated hyperbilirrubinemia [18], an idea that has been strengthened by some neuroimaging findings: with wider frontotemporal sulci, inter-hemispheric fissure, and lateral ventricular sizes in CT brain scan [21]; increased signal intensity in various areas in FLAIR MRI brain scan [22]; and decreased metabolism in various areas in 1H-MRS brain scan [23, 24]. Thus UCB high levels may have a potential role as an indicator in the dimensional assessment of severity and chronicity among patients with schizophrenia.

## Unconjugated bilirubin: a tool in the distinction between schizophrenia and bipolar disorder?

Schizophrenia, schizoaffective and bipolar disorders share some common symptoms but there aren't yet true biomarkers clearly separating those disorders. There is still a lot of controversy regarding schizoaffective disorder as an independent category and its relationship with schizophrenia and bipolar disorders [25]. We are aware at least of four different conceptual possibilities for schizoaffective disorder: a severe form of bipolar disorder in which episode-related psychotic symptoms fail to remit completely between mood episodes; a milder variant of schizophrenia in which mood symptoms are more prominent than usual; the co-occurrence of schizophrenia and bipolar disorder; or is it actually a third and different type of psychosis, completely autonomous from the bipolar and schizophrenia categories? [26].

We believe that UCB mean levels may contribute to some clarification of this controversy, as one of many potential biological markers, facilitating the distinction between these diagnostic categories and\or discriminating the dimensional severity among each of these psychotic conditions. In our perspective, searching for contributing factors in a biological domain and across a spectrum does not reflect a reductionist approach or the denial of the importance of individual vulnerabilities depending on affective, relational and communicational dimensions, which are determinants at a systemic level, in schizophrenia spectrum disorders.

## Unconjugated bilirubin: a study protocol for the psychosis spectrum

Our study will be an observational longitudinal study, with two assessments in 1 year time span, in order to achieve a better correlation between variables during the evolution of the patient's disorder and its respective treatment.

### Sample

After publishing a case report [17] we got interest in this topic, so we did a retrospective study and found a 0.1 mg\dL significant statistical difference (*p* ≤ 0.0001) between the UCB mean values of patients with schizophrenia (0.39 mg\dL with SD 0.16 mg\dL) patients vs. bipolar patients (0.29 mg\dL with SD 0.13 mg\dL) [14]. Using www.openepi.com software, with a confidence interval of 95%, and a beta value of 80%, we calculate a minimum sample size of 34 patients per group. Thus we expect to need no more than 70 individuals, composed by two different groups (35 patients with schizophrenia and 35 patients with schizoaffective disorder).

### Inclusion criteria

Age older than 18 but below 65 years old; understanding and signing informed consent; diagnosis (any type) of schizophrenia or schizoaffective disorder, according to ICD10 criteria [27]. Exclusion criteria: Any substance abuse detected on urine test; any organic brain disorder impairing protocol assessment; any hepatic, hemolytic, or cholestasis related condition detected on blood work. Scientific and Ethical approval: obtained at local Scientific and Ethical Boards.

### General variables

We would like to verify if exists any kind of influence on UCB mean levels by any of the following general variables: age; gender; fasting; exercise; occupation; education years; smoking (pack-year) [28]; use of contraceptive pill; body mass index (weight\height^2^); family history of psychiatric disorder; duration of psychiatric disorder (years since first diagnosis); treatment setting (outpatient or inpatient); mean duration of Psychiatric admission (days); metabolic syndrome comorbidity (diabetes, dyslipidemia, and\or hypertension), psychiatric medication (controlled through chlorpromazine 29, 30 and benzodiazepine 31 equivalents).

### Biochemistry study

Calculated UCB serum levels (UCB = Total Bilirubin – Direct Bilirubin); blood sample will be taken and all procedures will be readily made: blood collection: vacuum S-Monovette® Serum Gel Z/4.9 ml (Sarstedt AG&Co); analytic method: 2,4-dicloroanilina (DCA) photometry; laboratory hardware System: ABX Pentra 400® (Horiba Group); laboratory software system: SISLAB® (Glintt); blood work (hemogram, LDH, AST, ALT, GGT, HCV, and HBV); urine drug testing (cannabinoids, amphetamines, cocaine and heroin).

### Psychopathological instruments

Clinical Global Impression (CGI) [32]—for general clinical severity; and Positive and Negative Schizophrenia Scale (PANSS) [33]—for psychosis severity.

### Neuropsychological instruments

We will, for the first time ever, test any kind of correlation between UCB mean levels and the following neuropsychological changes, previously described in patients with schizophrenia [34]; Montreal Cognitive Assessment (MOCA) [35]—general cognitive assessment. Trail Making Test-A (TMT-A)—cognitive processing speed; Trail Making Test-B (TMT-B) [36]—executive functions; and Wechsler Adult Intelligence Scale Digital Span (WAIS-DS) [37]—attention and working memory.

### Psychosocial instruments

We will test, also for the first time, any kind of correlation between UCB mean levels and Personal and Social Performance (PSP) [38]—for social functioning assessment.

For the two patients group (35 patients with schizophrenia and 35 patients with schizoaffective disorder), all study quantitative variables will be summarized through descriptive statistics namely mean, median, standard deviation, range, and proportions. This analysis include: socio-demographics characterization as well psychosocial (PSP), psychopathological (CGI, PANSS), neuropsychological (MOCA, TMT, WAIS-DS), and biological variables (biochemical study including UCB). For a better control of confounding variables patients will be paired regarding their age, gender, and level of education. Regarding statistics we plan to use the Statistical Package for the Social Sciences (SPSS) software.

## Unconjugated bilirubin: another difference between schizophrenia and schizoaffective disorder?

In future studies we will try to contribute for a better understanding of biological aspects in patients with psychosis, and particularly the possible role of UCB as an indicator in the distinction among patients with schizophrenia and schizoaffective disease. Thus, as our main objective, we would like to test two different hypotheses:

1. Is UCB serum level a diagnosis indicator, with (diagnostic and nosological) categorical distinction potential, between groups of patients with different psychotic disorders (e.g., between schizophrenia vs. schizoaffective disorder)?
2. Is UCB serum level a severity indicator, with (psychopathological, neuropsychological, and psychosocial) dimensional distinction potential, among groups of patients with the same psychotic disorder (e.g., among patients with schizophrenia)?

We shall highlight that UCB mean levels have never been studied before in patients with schizoaffective disorder, nor have been correlated with neuropsychological and psychosocial variables in any kind of psychiatric patients. Thus our future studies will try to innovate, adding more data to the hypothesis of a categorical but also dimensional spectrum of psychosis: schizophrenia—schizoaffective disorder.

## Author contributions

JG: designed the project and wrote the article; FA-G: commented on the manuscript.

### Conflict of interest statement

The authors declare that the research was conducted in the absence of any commercial or financial relationships that could be construed as a potential conflict of interest.
